# 3D Quantitative and Ultrastructural Analysis of Mitochondria in a Model of Doxorubicin Sensitive and Resistant Human Colon Carcinoma Cells

**DOI:** 10.3390/cancers11091254

**Published:** 2019-08-27

**Authors:** Claudia Moscheni, Emil Malucelli, Sara Castiglioni, Alessandra Procopio, Clara De Palma, Andrea Sorrentino, Patrizia Sartori, Laura Locatelli, Eva Pereiro, Jeanette A. Maier, Stefano Iotti

**Affiliations:** 1 Department of Biomedical and Clinical Sciences “Luigi Sacco”, Università degli Studi di Milano, 20157 Milano, Italy; 2 Department of Pharmacy and Biotechnology, University of Bologna, 40127 Bologna, Italy; 3 Unit of Clinical Pharmacology, “Luigi Sacco” University Hospital, ASST Fatebenefratelli Sacco, 20157 Milan, Italy; 4 ALBA Synchrotron Light Facility, Carrer de la Llum 2-26, 08290 Cerdanyola del Vallès, Spain; 5 Department of Biomedical Sciences for Health, Università degli Studi di Milano, 20133 Milan, Italy; 6 National Institute of Biostructures and Biosystems, 00136 Roma, Italy

**Keywords:** mitochondria, multidrug resistance, doxorubicin, cellular metabolism, nanoscale imaging, soft X-ray cryo tomography, transmission electron microscopy

## Abstract

Drug resistance remains a major obstacle in cancer treatment. Because mitochondria mediate metabolic reprogramming in cancer drug resistance, we focused on these organelles in doxorubicin sensitive and resistant colon carcinoma cells. We employed soft X-ray cryo nano-tomography to map three-dimensionally these cells at nanometer-resolution and investigate the correlation between mitochondrial morphology and drug resistance phenotype. We have identified significant structural differences in the morphology of mitochondria in the two strains of cancer cells, as well as lower amounts of Reactive oxygen species (ROS) in resistant than in sensitive cells. We speculate that these features could elicit an impaired mitochondrial communication in resistant cells, thus preventing the formation of the interconnected mitochondrial network as clearly detected in the sensitive cells. In fact, the qualitative and quantitative three-dimensional assessment of the mitochondrial morphology highlights a different structural organization in resistant cells, which reflects a metabolic cellular adaptation functional to survive to the offense exerted by the antineoplastic treatment.

## 1. Introduction

Doxorubicin (DXR) is a powerful anthracycline antibiotic widely used to treat many human tumors. It frequently induces multidrug resistance (MDR), one of the most serious obstacles to successful anticancer chemotherapy. MDR is associated with the modulation of different biological events (i.e., cell cycle, apoptosis and DNA damage) [1] and the alteration of several cellular phenotypic features. MDR tumor cells often show the overexpression of membrane efflux proteins pumping antitumor drugs out of the cell with a consequent decrease of their accumulation and/or modification of their intracellular distribution.

Cancer cell metabolic state has received increasing attention over the last decade, mainly in relation to proliferation and specific metabolic alterations, and only recently it has been implicated in tumor drug resistance [2,3]. For long time the “Warburg Effect”, the most popular metabolic theory, has described an increase in glucose uptake and a predominant shift of metabolism from oxidative phosphorylation (OXPHOS) towards aerobic glycolysis as cancer hallmarks [4]. Nowadays, it is known that cancer cells are heterogeneous at the metabolic level, as well as functional, proliferative and differentiating potential. Although tumors appear mainly glycolytic due to the prevalence of cells with glycolytic features, other metabolic programs coexist [5] and become evident in particular under pharmacological treatment. A reversion of the Warburg effect in response to chemotherapy has been demonstrated in different chemoresistant tumor cells [6,7] as a possible strategy to cope with the increase of Adenosine triphosphate (ATP) demand by enzymes involved in DNA repair, drug efflux and detoxification.

Mitochondria are dynamic organelles that regulate cellular energy generation, calcium and redox homeostasis, and cell death. They also interfere with epigenetic aspects, with the degree of stemness and cell differentiation. Mitochondria exhibit continuous changes in shape, through fusion and fission mechanisms, or even by increasing size to form giant mitochondria, and alter the number or the shape of the cristae [8]. Moreover, the existence of a mitochondrial network (mt-network), in close contact with the cellular organelles and the cytoskeleton has been demonstrated [9].

Mitochondrial morphology reflects the cellular function of the organelles [10] and is continuously modified in response to the different functional requirements of the cell. A rapid and reversible change of the mitochondrion from the so-called ‘orthodox’ to ‘condensed’ conformations as well as a shift from fragmented state to tubular continuum of the mt-network were revealed upon activation of ATP synthesis by OXPHOS [11,12].

Cancer cells develop abnormal metabolic functions with consequent morphological alteration of the mitochondria or mt-network [13]. Nevertheless, the mitochondrial ultrastructural alterations in human tumors are not specific for any neoplasm. Regardless of tumor histogenesis, when visualized by transmission electron microscopy (TEM) the mitochondria can exhibit either a dense matrix (condensed pattern) or an electron-lucent matrix (orthodox pattern) associated with disarrangement and distortion of the cristae and partial or total cristolysis. It has been hypothesized that cells with dense mitochondria could be hypoxia-tolerant and, therefore, able to generate sufficient ATP from oxidative phosphorylation. Conversely, the hypoxia-sensitive cells unable to produce sufficient amounts of ATP by mitochondrial respiration could be characterized by orthodox mitochondria and cristolysis [14].

It is known that DXR treatment interferes with mitochondria inducing alterations in mitochondrial respiratory function and enzymatic activities [15], potentially promoting the occurrence of MDR. Recently, the effect of the cellular morphology on cellular proliferation and drug responsiveness was investigated in two different types of breast cancer cells [16] providing experimental evidence of the intimate correlation between cell morphology parameters and drug sensitivity.

In the present study we have elaborated, using soft X-ray cryo transmission tomography (cryo-SXT), 3D maps of vitrified DXR-sensitive and DXR-resistant cancer whole-cells with nanometer resolution, contributing to expand the knowledge about the correlation between mitochondrial morphology/function and MDR phenotype. Given the complexity of the MDR, a careful characterization of in vitro models could shed some light on the biological processes involved in chemoresistance etiology, and, therefore, lead to the development of new therapeutic strategies for its overcoming.

## 2. Results

### 2.1. Confocal Microscopy of LoVo-S and LoVo-R Mitochondria

Initially, we analyzed LoVo cells sensitive (LoVo-S) or resistant to DXR (LoVo-R) by confocal microscopy. Phalloidin and 4,6-diamidino-2-phenylindole (DAPI) were utilized to visualize the actin filaments and the nuclei, respectively. Antibodies against cyclophilin F were used to highlight the mitochondria.

**Figure 1:**
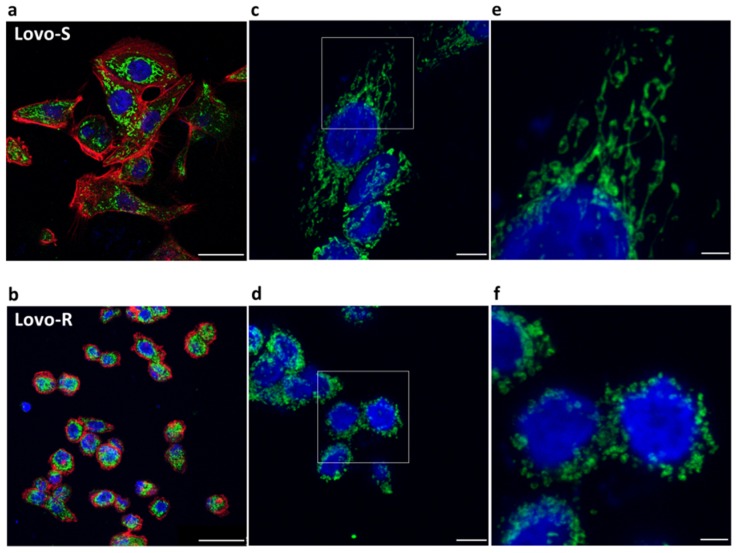
Confocal microscopy of LoVo-S and LoVo-R mitochondria. Representative photomicrographs of LoVo-S (**a**,**c**,**e**) and LoVo-R (**b**,**d**,**f**) mitochondria. Mitochondria were marked with anti cyclophilin F antibody and analyzed by confocal microscopy. Phalloidin (**a**,**b**) and DAPI were utilized to visualize the actin filaments and the nuclei, respectively. Right panel: zoom of the white square. Scale bar: 25 μm (**a**,**b**), 8 μm (**c**,**d**) and 3 μm (**e**,**f**).

As shown in Figure 1, LoVo-S (Figure 1a) exhibited an elongated flattened morphology with a central oval nucleus, while LoVo-R (Figure 1b) appeared smaller, with a round shape and a central round nucleus. Mitochondria were scattered in the cytosol both in LoVo-S and LoVo-R, but while LoVo-S displayed a complex mitochondrial network with elongated mitochondria (Figure 1c,e), LoVo-R revealed diffusely distributed round mitochondria (Figure 1d,f).

### 2.2. Ultrastructural Analysis

**Figure 2:**
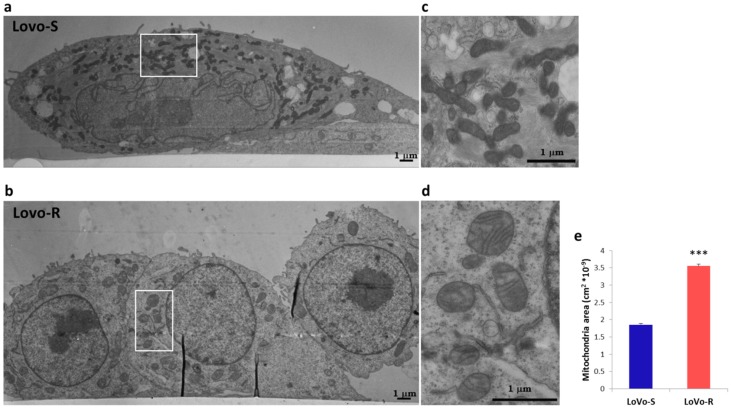
2D Ultrastructure analysis of LoVo-S and LoVo-R mitochondria by electron microscopy. Electron microscopy micrographs showing LoVo-S (**a**,**c**) and Lovo-R (**b**,**d**) cells cultured in a 2D-monolayer. LoVo-S are flattened cell adhering to the plastic dish and partially overlapping (**a**), while LoVo-R exhibit a round shape and a weak relationship with the substrate (**b**). A greater enlargement (**c**) of the outlined areas of the figure (**a**) that indicate LoVo-S mitochondria with preserved ultrastructure and electrondense matrix. A greater enlargement (**d**) of the outlined areas of the figure (**b**) that show mitochondria with a pale matrix, disarrangement of the cristae and moderate cristolysis. In (**e**) mitochondria area was manually calculated on randomly electron micrographs. Values are expressed as mean ± SEM and compared using the Student’s *t* test (*** *p* < 0.001).

As previously described [17], ultrastructural analysis by TEM confirmed the marked morphological differences between LoVo-S and LoVo-R (Figure 2). LoVo-S (Figure 2a) are larger than the LoVo-R (Figure 2b), have an irregular nucleus with dispersed chromatin and, in adhesion to the Petri dish, show an elongated shape with some overlapping cellular portions and intercellular junctions. Under the same experimental conditions, LoVo-R (Figure 2b) appeared roundish with a regular nucleus and dispersed chromatin. Electron microscopy also showed irregularly shaped and sized mitochondria with a dark, condensed matrix and large cristae in LoVo-S (Figure 2c). In LoVo-R mitochondria were more regular in shape and dimensions and exhibited a lucent matrix, fewer and disarrayed cristae and, sometimes, a modest cristolysis (Figure 2d). No evident mitophagy was detected in both cell lines. The evaluation of the mitochondria mean area, carried out by randomly acquired electron microphotographs, showed that these organelles were significantly larger in LoVo-R than LoVo-S (Figure 2e).

### 2.3. 3D Ultrastructural Quantitative Analysis of LoVo-S and LoVo-R Mitochondria by Synchrotron-Based Cryo-SXT

**Figure 3:**
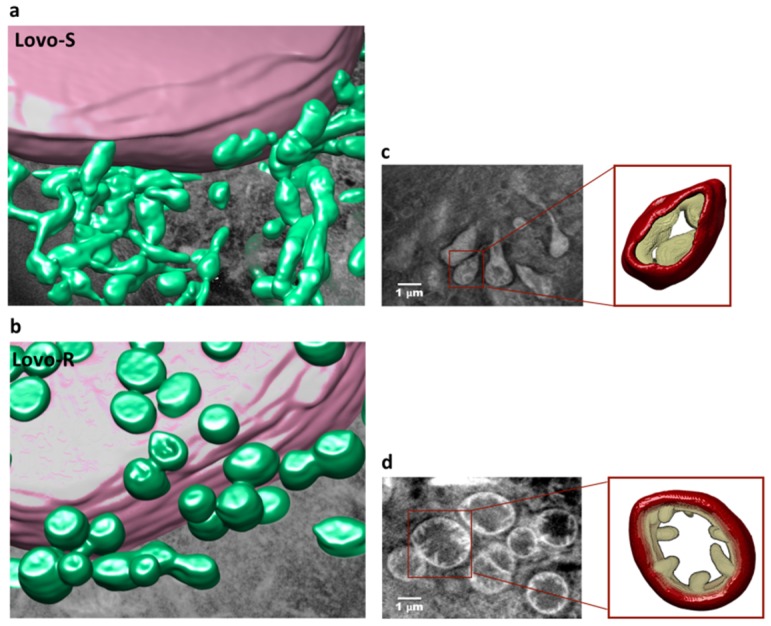
3D ultrastructural analysis of LoVo-S and LoVo-R mitochondria by synchrotron-based cryo-SXT. Color-coded manual segmentation of the surface boundaries (**a**,**b**) identifying the nucleus (violet) and mitochondria (green) of three-dimensional reconstruction of whole-cell volumes of LoVo-S and LoVo-R, obtained by cryo-SXT (pixel size 11.8 nm). In (**c**) one slice of the tomogram from the same volume region shown in (**a**) and 3D rendering of a single selected mitochondrion (right panel) of LoVo-S. One slice of the tomogram (**d**) from the same volume region showed in (**b**) and 3D rendering of a single mitochondrion (right panel) of LoVo-R.

Synchrotron-based cryo-SXT was carried out to perform a 3D ultrastructural quantitative analysis of mitochondria at nanoscale. From cryo-SXT reconstructions we obtained the 3D nano-rendering of the whole cells allowing virtual tour inside the cell (Videos S1 and S2) with the opportunity to extract quantitative information such as volume, shape and linear absorption coefficient (LAC) of mitochondria. Figure 3 shows elongated mitochondria in LoVo-S and almost spherical in LoVo-R. Furthermore, in LoVo-S the mitochondria appeared as a network of interconnected organelles, while they seemed isolated and fragmented in LoVo-R. To highlight these differences, the 3D rendering of representative volume regions both for LoVo-R and -S cells was reported in Figure 3 (panels a and b) together with the 3D rendering of a single representative mitochondrion (panels c and d). Figure 3c and d show that the shape of the cristae, tube-like extensions of the mitochondrial inner membrane, was different in the mitochondrion of LoVo-R and LoVo-S. In LoVo-R the cristae were thin and protrude into an expanded and translucent matrix, while in LoVo-S there were fewer cristae embedded into the inner mitochondrial membrane. These morphological differences are relevant because cristae shape has a role in the assembly and structure of OXPHOS system [18,19] with important consequences on ATP synthesis.

To further investigate the differences in mitochondrial shape in LoVo-R and LoVo-S, we calculated the Fractional Anisotropy (FA) of each mitochondrion [20,21]. Anisotropy is the property of being directionally dependent, hence FA describes the degree of anisotropy ranging between 0 and 100. When the FA = 0, the mitochondrion does not have a preferential direction but it is equally distributed along the three axes (*λ_1_, λ_2_, λ_3_* eigenvalues). By contrast, the FA = 100 means the mitochondrion has a preferential direction and it is limited along the other two axes. Specifically, the eigenvalues of the diffusion tensor allow calculating the FA (see Equation (5) in the Materials and Methods) [22].

**Figure 4:**
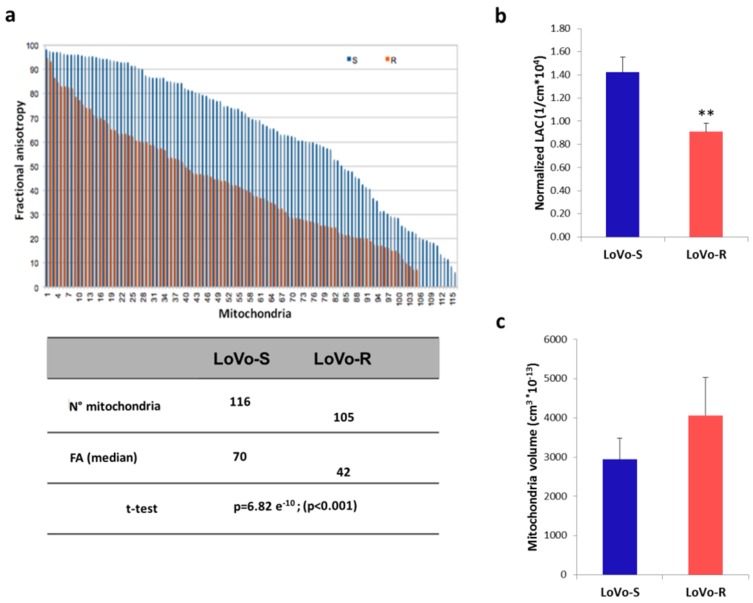
Fractional Anisotropy and LAC of 3D LoVo-S and LoVo-R mitochondria. (**a**) illustrates the average difference in FA between mitochondria of LoVo-S and Lovo-R; in the table, we reported the two median values of FA and the results of comparison using the Student’s *t* test. In (**b**) Histogram reports the estimated normalized LAC for mitochondria of 7 LoVo-S and 7 LoVo-R. Values are expressed as mean ± standard error of the mean and compared using the Student’s *t* test (** *p* < 0.01); in (**c**) histogram reports the measured volume of mitochondria in 7 LoVo-S and 7 LoVo-R. Values are expressed as mean ± SEM and compared using the Student’s *t* test. No statistically differences are obtained.

In Figure 4a, we report the FA values (%) of 116 and 105 mitochondria, respectively belonging to LoVo-S and LoVo-R. The FA median value of LoVo-S mitochondria (70%) confirmed that the shape is anisotropic (elongated shape), while the FA median value of the LoVo-R mitochondria (40%) revealed their more isotropic shape (almost round shape) with a high degree of statistical significance (*p* < 0.001).

Concerning the mitochondrial matrix density, Figure 4b showed a statistically significant decrease (*p* < 0.01) of the normalized LAC of the mitochondria of LoVo-R in respect to LoVo-S, suggesting that mitochondria had a less condensed matrix in LoVo-R than in LoVo-S. Finally, no significant difference was revealed in the mitochondria total volume between the two cell lines (Figure 4c).

### 2.4. Mitochondrial Content and Activity

**Figure 5:**
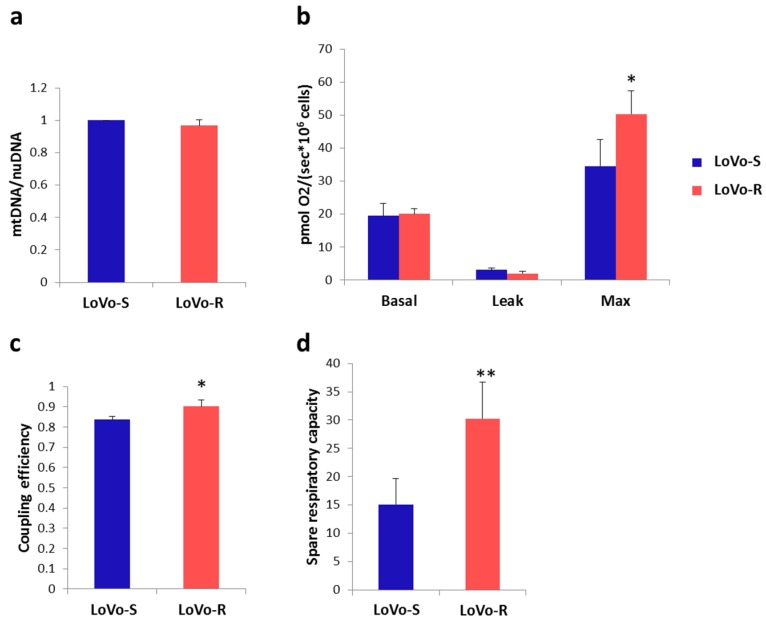
Oxygen consumption in LoVo cells. (**a**) mtDNA quantification by quantitative Polymerase Chain Reaction (qPCR) on total DNA extracted from LoVo-S and LoVo-R (*n* = 3 experiments); (**b**) Oxygen fluxes following oligomycin (0.5 μm), stepwise titration of the chemical uncoupler FCCP (0.5 μm), and rotenone (0.5 μM)/antimycin A (2.5 μm) were recorded; (**c**) Mitochondrial coupling efficiency representing the respiratory fraction that leads to ATP synthesis (*n* = 5 experiments); (**d**) Spare respiratory capacity as the result of the subtraction of the basal respiration from maximal respiration rates.

LoVo-S and -R showed identical mitochondrial content, as measured by quantification of mtDNA levels (Figure 5a). Basal mitochondrial respiration was similar in LoVo-R and LoVo-S (Figure 5b). The inhibition of ATP synthase with oligomycin induced a state of minimum respiration that did not differ between the two cell types. Even though there were no differences in cellular respiration at the basal level, coupling efficiency, which measures the proportion of nutrient energy that is converted into ATP, was higher in LoVo-R than LoVo-S (Figure 5c). Furthermore, LoVo-R performed better in sustaining the increased workload driven by the addition of the protonophore Carbonyl cyanide-4-(trifluoromethoxy) phenylhydrazone (FCCP) (Figure 5b).

Consistently, spare respiratory capacity was enhanced in LoVo-R (Figure 5d), thus assessing the capability of the respiratory chain to fit an energetic demand, as well as the maximal respiration achievable by the cell.

**Figure 6:**
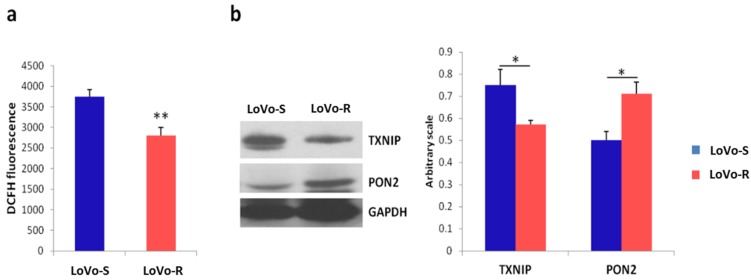
ROS production in LoVo cells. (**a**) ROS were measured by monitoring the emission at 529 nm of the DCFH dye in LoVo cells (*n* = 3 experiments); (**b**) Representative immunoblot for TXNIP and PON2. GAPDH was used as a control of loading. Densitometric quantification is provided. Values are expressed as mean ± SEM and compared using the Student’s *t* test (* *p* < 0.05, ** *p* < 0.01).

Mitochondria are major contributors to the production of cellular ROS and ROS-mediated signaling controls mitochondrial dynamics [23]. On these bases, we measured ROS production and found higher amounts of ROS in LoVo-S than in LoVo-R (Figure 6a). These differences are partly due to the downregulation of the pro-oxidant thioredoxin-interacting protein (TXNIP) associated with the upregulation of the anti-oxidant enzyme paraoxonase (PON2) in LoVo-R vs LoVo-S, as detected by western blot (Figure 6b). More studies are in progress to fully define the redox balance in LoVo-R and LoVo-S.

## 3. Discussion

With cancer progression, mutations and epigenetic drift promote the emergence of genetically diverse tumor cells with distinct molecular signatures. Moreover, harsh micro-environmental conditions during and after chemotherapeutic treatment fuel cellular adaptive responses to favor cell survival [24,25]. These events generate cellular heterogeneity within the tumor, thus feeding drug resistance, which occurs in virtually every type of anti-cancer therapy and is one of the major obstacles in the treatments of malignancies.

DXR, which is used for the treatment of a wide variety of cancer types, makes no exception. DXR resistance is common and understanding its cellular and molecular basis represents a challenge. We here utilize LoVo colon carcinoma cells sensitive or resistant to DXR. We have previously shown that silencing the channel/enzyme TRPM7 switches the phenotype of LoVo-S to one more similar to LoVo-R [17]. Moreover, in LoVo-R we have demonstrated a higher concentration of total intracellular magnesium (Mg) than in LoVo-S [26,27], and this is associated with a different spatial distribution and, specifically, a significant accumulation in proximity to the plasma membrane [28,29]. These findings hint to a direct relationship between Mg homeostasis and subcellular localization and efflux pump function, considering that: (i) MDR proteins belong to the family of ATP-binding cassette which transports a substrates across biological membranes in an energy-dependent manner [30] and (ii) MgATP^2–^ is the active species in enzyme binding, in the cellular active transport and the form responsible for the energy production [31].

Mg, crucial in all the metabolic pathways, is mainly stored within the mitochondria [32] and Mrs2, a mitochondrial Mg transporter, is upregulated in a multidrug-resistant gastric cancer cell line [33]. It is also interesting that the overexpression of the mitochondrial Mg channel Mrs2 increases total cellular Mg concentration and decreases sensitivity to apoptosis [34]. All these studies support the relevant role of mitochondria in chemoresistance [35,36]. Accordingly, DXR treatment causes adaptive responses through the mitochondria [37], potentially contributing to the occurrence of MDR.

The direct relationship that links mitochondrial shape and function [38] bolsters the need to visualize and describe their shapes in detail. In this study we have investigated the salient morphological characteristics of these pivotal organelles in an in vitro model of MDR. To get the complete 3D structure of the entire cell in its native state, the data obtained by light and transmission electron microscopy were complemented with cryo-SXT measurements. It is noteworthy that the X-ray tomograms are fully compatible with the higher resolution TEM images from cell thin sections. This correlation is important because it validates the structures observed by both methods and, furthermore, it allows expanding the results from light and transmission electron microscopy to the three-dimensional picture of whole cell and its mitochondria.

While LoVo-S and LoVo-R possess identical mtDNA levels, clear morphological differences emerged. By electron microscopy we observed that the mean mitochondrial area was significantly smaller in LoVo-S than in LoVo-R. Through the three-dimensional cryo-SXT reconstructions we calculated the total mitochondrial volume and no significant difference was revealed between the two cell lines. This divergence between area and volume data is due to the fact that the mitochondria exhibit very different 3D shapes in LoVo-S and -R, a fact that was partially hinted in the TEM thin sections.

The irregular profile of LoVo-S mitochondria, observed by confocal and electron microscopy, was confirmed by their 3D reconstruction, which highlights the presence of elongated branched mitochondria in close proximity to each other to form a complex network. Conversely, LoVo-R 3D reconstruction shows “simple”, fragmented round mitochondria. These qualitative observations have been validated and quantified by the evaluation of the Fractional Anisotropy which demonstrates a greater morphological difference of LoVo-S mitochondria compared to LoVo-R.

A wealth of recent evidence indicates a close relationship between energy production and mt-network organization [38]. Although the exact mechanism of defective mitochondrial dynamics in cancer development is not known, it has been observed that excessive mitochondrial fission is associated to many tumor cells survival [39]. Moreover the resistance to anticancer therapies relies on the metabolic adaptation of cancer cells [40] and mitochondrial shape might become a key mechanism through which tumor cells promote the metabolic shifts required for resistance to chemotherapy.

Another crucial aspect to consider is mitochondrial communication, which occurs via the release of soluble signaling molecules, among which ROS. Since the diffusion distance limits the communicative capacity, the lack of an interconnected network in LoVo-R cells could induce mitochondria to act as discrete organelles, preventing the establishment of a diffuse cellular apoptotic mechanism [41] stimulated by DXR treatment and providing a possible thrust towards MDR. In addition, LoVo-R have lower amounts of ROS than LoVo-S, and this might further impair mitochondrial communication. The reduction of ROS in LoVo-R is, in part, ascribed to the upregulation of the antioxidant enzyme PON2 and the downregulation of the pro-oxidant TXNIP. More studies are necessary to fully understand the mechanisms responsible for the different levels of ROS in LoVo-R and LoVo-S.

Electron microscopy ultrastructural analysis has also revealed a different degree of condensation of the mitochondrial matrix between sensitive and resistant cells to DXR. LoVo-R had mitochondria with an orthodox configuration, characterized in cross-section by thin cristae filling an expanded and more translucent matrix, while mitochondria of LoVo-S cells exhibited a mitochondrial morphology referred to as the condensed configuration with a dark, condensed matrix and fewer translucent cristae. The estimation of the mitochondrion LAC, carried out on the tomographic reconstructions, showed a significant difference of the mitochondrial matrix density between the two cell lines, corroborating the qualitative TEM ultrastructural observation on the two-dimensional thin sections.

A close coupling between mitochondrial ultrastructure and energetic state has been documented decades ago [11] and several authors confirm that stimulation of respiration induces the condensed conformation in the mitochondria [12,42]. Also, some recent modeling-based studies have provided insights into how mitochondrial morphology could contribute to bioenergetic function [19]. Nevertheless, different mitochondrial responses in different cell lines, especially if neoplastic, have been described following inhibition of glycolysis and/or OXPHOS stimulation [43].

Considering that we are at the beginning of understanding the dualities of mitochondrial functions in cancer progression and that mitochondrial metabolism could be either advantageous or detrimental for cancer cells, it would be important to unravel the mechanism of MDR.

Our data do not show a direct correlation between mitochondria matrix condensation and OXPHOS activity levels since the basal mitochondrial respiration was equivalent in LoVo-S and -R. However, the coupling efficiency and the spare respiratory capacity were significantly higher in LoVo-R than in LoVo-S. Thus, while sensitive cells have almost no mitochondrial reserve capacity, resistant cells have a reservoir of mitochondrial function which could allow metabolic adaptation useful to cope with the stress induced by the antineoplastic treatment. The ability of LoVo-R to sustain the increased workload and the extra-amount consumption of ATP, caused by the implemented cellular activities carried out to resist chemotherapy (i.e., increasing of drug efflux and inactivation, DNA damage repair, expression of anti-apoptotic genes and activation of cellular survival signaling), could contribute to the development of the MDR in these cells.

It has been demonstrated that the condensation of the mitochondrial matrix could correlate with the decrease in the mitochondrial membrane potential [44]. Moreover, when the condensed configuration is induced, a greater quantity of cytochrome C faces the space between the inner and outer membrane of the mitochondrion, thus impacting on the susceptibility of cells to apoptosis [44]. Therefore, the mitochondrial condensed matrix of LoVo-S could lead to cytochrome c exposure resulting in high sensitivity to apoptotic stimuli such as DXR.

Moreover cristae remodeling enhances mobilization of cytochrome C stores [45], suggesting that also LoVo cells cristae structures could differently impact on apoptosis. The resolution of our tomographies allowed three-dimensional reconstructions of mitochondrial cristae of LoVo-S and LoVo-R, highlighting a different mitochondrial ultrastructural organization. An in-depth future evaluation of all these aspects will be necessary to better clarify the mechanisms underlying the establishment of the MDR in LoVo.

## 4. Materials and Methods

### 4.1. Cell Culture

Human colon cancer LoVo cells sensitive (LoVo-S) or resistant to DXR (LoVo-R) were cultured in DMEM containing 10% fetal bovine serum and 2 mM glutamine, at 37 °C and 5% CO_2_ [17]. To maintain DXR resistance, LoVo-R were routinely treated with DXR (1 µg/mL). DXR was removed from culture media 5 days before performing the experiments to better compare LoVo-S and LoVo-R.

### 4.2. DNA Quantification

Mitochondrial DNA (mtDNA) from LoVo sensible and resistant cells, was quantified as previously described with slight modifications [46,47]. In brief, total DNA was isolated using phenol-chloroform extraction and the mtDNA content was quantified by qPCR by using specific primers for human mtDNA (ND1), and nuclear 28S ribosomal subunit. Primer sequences are listed below:

hND1:

Fw: 5′-CCCTAAAACCCGCCACATCT-3′

Rev: 5′-TAGAAGAGCGATGGTGAGAGCTA-3′

h28s:

Fw: 5′-TTAAGGTAGCCAAATGCCTCG-3′

Rev: 5′-CCTTGGCTGTGGTTTCGCT-3′

### 4.3. Reactive Oxygen Species (ROS) Evaluation

ROS were measured using 2′-7′-dichlorofluorescein diacetate (DCFH, cat. no. 35845, Sigma-Aldrich, St. Louis, MO, USA). After 48 h of culture into black bottomed 96-well plates (Greiner Bio-one, Frickenhausen, Germany), LoVo cells were washed with PBS and exposed to DCFH (20 μM). The emission at 529 nm of the DCFH dye was detected by the GloMax^®^-Multi Detection System (Promega, Madison, WI, USA). Three independent experiments were performed. Data are shown as the mean ± standard error of the mean (SEM).

### 4.4. Protein Isolation and Western Blotting

After 48 h from seeding, LoVo cells were lysed as previously described [17]. Cell extracts (100 μg/lane) were separated on SDS-PAGE and transferred to nitrocellulose sheets at 400 mA for 2 h at 4° C. The membranes were probed using antibodies against thioredoxin interacting protein (TXNIP), paraoxonase 2 (PON2) (Thermo Fisher Scientific, Waltham, MA, USA) and GAPDH (Santa Cruz Biotechnology, Inc., Dallas, TX, USA). After the incubation with horseradish peroxidase (HRP)-conjugated secondary antibodies, immunoreactive bands were detected by the SuperSignal Chemiluminescence Kit (Thermo Fisher Scientific) and revealed by the SuperSignal chemiluminescence kit (Pierce-Thermo Fisher Scientific, Waltham, MA, USA). Three independent experiments were performed. Densitometric analysis was performed by the ImageJ software (https://imagej.nih.gov/ij/).

### 4.5. Confocal Microscopy

Cells were seeded on coverslips. After 24 h of culture, the cells were washed with PBS, fixed in 4% paraformaldehyde/ 2% sucrose in PBS for 10 min at room temperature and washed with PBS. The samples were permeabilized with 0.3% Triton and incubated at room temperature with anti-cyclophilin F immunopurified IgGs (Thermo Fisher Scientific). After extensive washing, the samples were incubated with secondary Alexa Fluor 488 antibody (Invitrogen, Thermo Fisher Scientific), rhodamine-phalloidin and DAPI (Sigma-Aldrich). The cells were mounted with ProLong™ Gold Antifade Mountant (Invitrogen, Carlsbad, CA, USA) and images were acquired using a 40× objective in oil by a SP8 Leica confocal microscope (Leica, Wetzlar, Germania).

### 4.6. Transmission Electron Microscopy (TEM)

For ultrastructure analysis cells were grown to near confluence and fixed overnight at 4 °C in 2.5% glutaraldehyde in 0.1 M cacodylate buffer (pH 7.4) on a Nunc Sylgard coated Petri dish (Thermo Fisher Scientific). After postfixation with 1% osmium tetroxide at 0 °C for 30 min, the 2D-monolayer cultures in situ on Petri were stained with 2% aqueous uranyl acetate, dehydrated with ascending concentration of ethanol at 4 °C and embedded in Epon-Araldite resin. Ultrathin sections, obtained with a Leica Supernova ultramicrotome (Reichert Ultracut E and UC7; Leica Microsystems, Wetzlar, Germany), were stained with lead citrate and observed with a Zeiss EM10 electron microscope (Carl Zeiss, Oberkochen, Germany). Mitochondria mean area of LoVo-S (*n* = 106) and LoVo-R cells (*n* = 109) was measured manually using the Image Pro-Plus software (version 6.0) (Media Cybernetics, Inc., Washington, WA, USA) on randomly acquired electron micrographs.

### 4.7. Soft X-ray Cryo-Tomography

The cryo-SXT is an imaging technique able to produce 3D maps of vitrified whole-cell samples at nanometer-resolution, allowing to investigate the cells as close as possible to their native state, without staining, sectioning or using enhancing agents [48].

LoVo-R and LoVo-S cells were seeded onto gold quantifoil R 2/2 holey carbon-film microscopy grids at a concentration of 1 × 10^4^ cell/cm^2^. Cells were incubated at 37 °C in 5% CO_2_ for 24 h. The samples were then gently rinsed with 100 mM ammonium acetate for two times, in order to remove salts and trace metals from the medium. Cells were frozen-hydrated by a rapid plunge freezing in liquid ethane bath cooled with liquid nitrogen using a Leica EM GP robot. As vitreous ice upon the samples causes X-ray absorption, excess water is removed before plunge freezing via blotting in order to obtain a total ice thickness well below 5 µm. Frozen grids were transferred under cryogenic conditions into the full field soft X-ray transmission microscope of the Mistral beamline at the ALBA-Light Source [49], where soft X-ray cryo transmission tomography measurements of whole frozen hydrated cells were performed. The cryogenic conditions were maintained during all the experiment.

Cryo-SXT was performed at 520 eV to optimize the contrast between the carbon-rich organelles membranes and the water-rich cytoplasmic solutions. For each cell, a tilt series was acquired using an angular step of 1 degree on a 140 degrees angular range.

To acquire the data sets a zone plate objective with an outermost zone width of 40 nm was used. The effective pixel size in the images was 11.8 nm. No radiation damage was detected at our spatial resolution. Each transmission projection image of the tilt series was normalized using flat-field images of 1 s acquisition time. The tilt series was manually aligned using eTomo in the IMOD tomography software suite [50]. In order to decrease as much as possible the deviations from an ideal rotation that creates artefacts in the reconstructed tomograms, the rotation of gold nano-beads was followed.

According with the Beer−Lambert law, the transmission *T*(*x*,*y*) is given by:

(1) $$T\left(x,y\right)=\frac{I\left(x,y\right)}{I_{0}\left(x,y\right)}=e^{\left(-\int µ\left(x,y,E_{0}\right)tdt\right)}=e^{\left(-\int {µ}_{m}\left(x,y,E_{0}\right)\rho tdt_{m}\right)},$$

where, *I*_0_ is the incident beam intensity, *I* is the transmitted intensity by the sample, *μ(E*_0_*)* is the X-ray LAC at incident energy *E*_0_, *μ_m_(E*_0_*)* = *μ(E*_0_*)*/*ρ* is the mass absorption coefficient at the same energy, *ρ* is the matrix density, *x* and *y* are the coordinates in the transversal plane at the sample position and the integral is extend through all the sample thickness. All the transmission tilt series have been converted in absorbance using ImageJ by applying the following expression:

(2) $$µ\left(E_{0}\right)t=-ln\left(T\right).$$

The absorbance tilt series were finally reconstructed with TomoJ [51], a plugin of ImageJ [52] using the ART iterative-algorithms with 15 iterations and a relaxation factor of 0.01.

### 4.8. Segmentation and Calculation of Mitochondria Density and Volume

The visualization and segmentation of the final volumes of mitochondria were carried out in Chimera software [53]. The voxel signal in the final reconstruction is the value of the LAC:

(3) $$µ\left(x,y,z\right)={µ}_{m}\left(x,y,z\right)\rho \left(x,y,z\right),$$

where *x*, *y*, *z* are the coordinates of the selected voxel within the volume. The LAC is linear with both the biochemical composition and the concentration of the considered elements. The measured LAC values are in good agreement with those reported by [54] (Nave et al.) The average LAC values and the corresponding standard deviation were calculated for approximately 15 to 20 mitochondria of seven LoVo-S and seven LoVo-R cells.

In order to compensate deviation of the measured LAC from different tomographies due to background variations and reconstruction artefacts, we normalized the estimated LAC values performing a calibration on the gold nano-beads that are used for alignment purposes (BBI Solution, 200 nm diameter). The corresponding normalized LAC values are reported in Figure 4b. We also calculated the mitochondrion volume using the following formula:

(4) $$Volume_{mitocondrion}=numberofvoxel\times voxeldimension.$$

For each cell, the mean volume value and the standard deviation were then obtained.

### 4.9. Fractional Anisotropy

The Fractional Anisotropy (FA) was calculated for all segmented mitochondria of both LoVo-S and LoVo-R cells. Each segmented mitochondrion was imported into the MATLAB software and the eigenvalues *λ*_1_, *λ*_2_, *λ*_3_ were calculated through the “regionprops3” command. The FA has been calculated by implementing the following formula [55]:

(5) $$FA=\sqrt{\frac{3}{2}}\frac{\sqrt{\left(\lambda_{1}-\overset{´}{\lambda }\right)+\left(\lambda_{2}-\overset{´}{\lambda }\right)+\left(\lambda_{3}-\overset{´}{\lambda }\right)}}{\sqrt{\lambda_{1}^{2}+\lambda_{2}^{2}+\lambda_{3}^{2}}}.$$

### 4.10. Oxygen Consumption Measurement

Respiratory chain functionality was evaluated as previously described [56,57] using the O2K oxygraph chambers. Briefly, 1 × 10^6^ of viable cells, grown in standard conditions, were transferred into oxygraph chambers (Oroboros Instruments, Innsbruck, Austria) at 37 °C in the respiration medium MiR06 (pH 7.1). Routine respiration was acquired when the signal of oxygen consumption was stable, while the leak respiration was measured after the addition of oligomycin (0.5 µM). By stepwise titration (0.5 μM each step) of the uncoupler carbonyl cyanide-4-(trifluoromethoxy) phenylhydrazone (FCCP) the release of the proton gradient was induced until maximum respiration was reached. The residual oxygen consumption was evaluated blocking mitochondrial respiration by the addition of 0.5 μM rotenone and 2.5 μM antimycin A (AA).

Oligomycin, FCCP, rotenone, and antimycin A are membrane-permeable and directly exert their effects. Malate and pyruvate feed electrons into complex I of the respiratory chain and they are impermeable to the intact plasma membrane. Thus, Thus, the integrity of the plasma membrane was tested by the initial addition of pyruvate (10 mM), malate (2 mM). The subtraction of the residual oxygen consumption subtracted from each steady state was used to correct the oxygen fluxes.

### 4.11. Statistical Analysis

Statistical significance was determined using the Student’s *t* test and set as following: * *p* < 0.05, ** *p* < 0.01, *** *p* < 0.001.

## 5. Conclusions

We have identified significant differences in the morphology of mitochondria in LoVo-S vs LoVo-R. We hypothesize that lower amounts of ROS in LoVo-R and the absence of an interconnected mitochondrial network might impair mitochondrial communication. Also, the qualitative and quantitative three-dimensional assessment of mitochondrial morphology in LoVo-R and -S in a condition as close as possible to their living hydrated state highlights mitochondrial morphological features in resistant cells which might be important in driving their adaptation to cope with the stress induced by the antineoplastic treatment.

## Supplementary Materials

The following are available online at https://www.mdpi.com/2072-6694/11/9/1254/s1, Video S1: StackLoVoS.mp4, Virtual stack of soft X-ray cryo transmission tomography of LoVoS cell (pixel size 12 nm), Video S2: StackLoVoR.mp4, Virtual stack of soft X-ray cryo transmission tomography of LoVoR cell (pixel size 12 nm).
